# Siamese Transformer-Based Building Change Detection in Remote Sensing Images

**DOI:** 10.3390/s24041268

**Published:** 2024-02-16

**Authors:** Jiawei Xiong, Feng Liu, Xingyuan Wang, Chaozhong Yang

**Affiliations:** 1 College of Computer Science, Xi’an Polytechnic University, Xi’an 710600, China; jw-xiong@stu.xpu.edu.cn (J.X.); xingyuanwang@stu.xpu.edu.cn (X.W.); 2 National Time Service Center, Chinese Academy of Sciences, Xi’an 710600, China; ycz@ntsc.ac.cn

**Keywords:** Siamese transformer network, building change detection, difference comparison, remote sensing image

## Abstract

To address the challenges of handling imprecise building boundary information and reducing false-positive outcomes during the process of detecting building changes in remote sensing images, this paper proposes a Siamese transformer architecture based on a difference module. This method introduces a layered transformer to provide global context modeling capability and multiscale features to better process building boundary information, and a difference module is used to better obtain the difference features of a building before and after a change. The difference features before and after the change are then fused, and the fused difference features are used to generate a change map, which reduces the false-positive problem to a certain extent. Experiments were conducted on two publicly available building change detection datasets, LEVIR-CD and WHU-CD. The F1 scores for LEVIR-CD and WHU-CD reached 89.58% and 84.51%, respectively. The experimental results demonstrate that when utilized for building change detection in remote sensing images, the proposed method exhibits improved robustness and detection performance. Additionally, this method serves as a valuable technical reference for the identification of building damage in remote sensing images.

## 1. Introduction

Change detection in remote sensing images refers to the identification of changes in objects or phenomena of interest in a scene by comparing multitemporal remote sensing images captured at different times in the same geographic area. This technique can be applied to various application domains, such as urban planning, disaster management, agricultural surveys, and environmental monitoring.

Most early change detectors in remote sensing images were designed with the help of manual features and supervised classification algorithms. However, deep learning technology has developed rapidly in recent years, especially deep convolutional neural networks (CNNs), which can learn data representations with multiple levels of abstraction [1] and have been widely used in computer vision [1] and remote sensing [2]. Thus, many current change detection algorithms are based on deep learning [3] and demonstrate better performance than that of traditional methods. These deep learning methods can be broadly categorized into two groups: metric-based methods [4,5,6] and classification-based methods [7,8,9,10].

Metric-based approaches identify changes by assessing the parametric distance between dual-temporal data. These methods need to learn a parameterized embedding space, where similar or unchanged samples are clustered together in the embedding vector space, while different or changing samples are spread further apart. This approach helps to clearly distinguish samples of different categories or characteristics in the embedding space. The embedding space can be learned through deep Siamese fully convolutional networks (FCNs) [4,5]; “Siamese networks” refer to neural network architectures that learn to differentiate between two different inputs. The name “Siamese” originates from the concept of Siamese twins, as these networks are designed to share the same weights and architecture, effectively creating a mirrored structure. By simultaneously processing two inputs and then comparing their outputs, Siamese networks can determine the similarity or dissimilarity between the inputs. Siamese networks have been widely used in various applications, including signature verification, facial recognition, and similarity-based tasks such as image and text matching. In the context of remote sensing and image analysis, Siamese networks have been applied to tasks such as change detection, where they can effectively compare two images captured at different times to identify the differences between them or changes in the observed scene. This refers to a system comprising two identical networks with shared weights. Each network autonomously produces feature maps for individual temporal images. The metric comparing the features of each point pair is employed to determine if a change has occurred. The difference module employs an optimal metric method by comparing bitemporal images, and the Euclidean distance between the feature maps of the Siamese networks is calculated on a per-pixel basis, generating a difference feature map.

Classification-based methods determine the type of change by categorizing features extracted from two different time periods. Typically, a common approach involves assigning a change score to every location within an image, and the scores of changed locations are higher than those of nonchanged locations. CNNs have been widely used to extract feature representations of images [7,8,9,11]. By combining classification-based methods with the feature extraction capabilities of convolutional neural networks (CNNs), it is possible to more accurately identify and understand categories of variation in bichronological data.

In high-resolution remote sensing images, building changes can be monitored at a detailed scale. Deep convolutional networks [12] (ConvNets) are capable of extracting powerful discriminative features from these images. Currently, cutting-edge change detection methods primarily rely on deep convolutional networks. Since the capture of long-range context information in space and time is critical for identifying relevant changes in multitemporal images, the latest change detection research has focused on increasing the receptive field of change detection models [13,14]. Hence, change detection models [15] have been introduced. These models incorporate layered convolutional structures, dilated convolution operations [16,17], and a range of attention mechanisms [18,19]. In line with the transformer [20] architecture introduced in natural language processing, a variety of designs have been suggested for a range of computer vision tasks. These tasks encompass image classification and image segmentation, with examples such as the vision transformer (ViT) [21] and segmentation transformer (SETR) [22]. Transformer networks exhibit larger effective receptive fields than deep convolutional neural networks, providing stronger global context modeling ability than that of a CNN between any pair of pixels in an image. Dosovitskiy et al. introduced the ViT for image classification. The authors, inspired by the design of the transformer in natural language processing, divided each image into several small patches with linear embeddings. These patches were then fed into the standard transformer with position embeddings, resulting in a remarkably strong performance on ImageNet [23]. In the realm of semantic segmentation, Zheng et al. proposed the SETR to demonstrate the viability of employing a transformer for this task. However, these models usually generate more redundant and false-positive outcomes; while the ViT has demonstrated strong performance in vision tasks, the limitations of outputting single-scale low-resolution features hinder the model’s further enhancement in the domain of change detection in remote sensing images. Therefore, it is crucial to explore methods for extracting superior multiscale features within an appropriate range for effective building change detection, as features at different scales can better process building boundary information.

To address the problem of rough boundary information processing and the presence of errors in the task of building change detection by the above methods, a Siamese transformer network structure utilizing a difference module is suggested for detecting changes in remote sensing images. This method mainly contributes to the following three points.

(1) A Siamese layered transformer feature extractor is used to better extract multiscale features of buildings in dual-temporal remote sensing images;
(2) A metric-based feature difference module is used to calculate the multiscale feature differences of buildings;
(3) A lightweight decoder is used to fuse multilevel feature differences of buildings and predict whether these buildings have changed in remotely sensed images.

## 2. Algorithm Design

### 2.1. Overall Architecture

**Figure 1:**
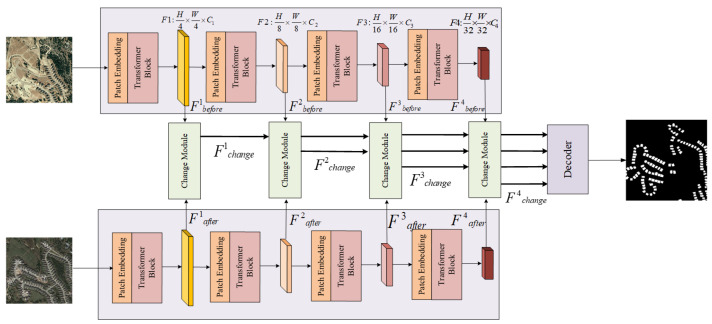
Overall network architecture.

Given a pair of dual-temporal remote sensing images as the model input, a layered Siamese transformer feature extractor generates multiscale features from the target building, and the input of the difference module at the i-th layer corresponds to the feature associated with that layer. The feature difference module calculates the features. The difference feature map for the building before and after a change are derived by calculating the Euclidean distance between pixels in the map. Ultimately, the change map is acquired following processing by the decoder, as shown in Figure 1.

### 2.2. Encoder

First, the input remote sensing image is segmented into nonoverlapping patches. Here, each patch can be considered an ordered input unit, whose feature is set to the concatenation of the RGB values of the original pixel. In this paper, each patch is set to be smaller because it is more conducive to the intensive task of detecting buildings. In actual operation, the 4 × 4 patch is used as the basic unit of sequence input. Therefore, the feature dimension of each patch is 4 × 4 × 3 = 48, and these original features can be projected to any dimension after passing a linear projection layer.

#### 2.2.1. Patch Merging Block

The idea of patch merging is to cut the input image into multiple patches and merge the patches into a larger representation. This process is performed at multiple levels to extract high-level semantic information in remote sensing images. Patch merging can capture the local information of remote sensing images at multiple scales, thus helping the model learn richer and more discriminative feature representations. The spatial resolution in remote sensing refers to the level of detail or granularity of the information that can be captured in an image. It is typically expressed in terms of the size of the smallest discernible image feature, which is often measured in meters per pixel. The spatial resolution influences the amount of contextual information that is available to the model. Higher-resolution images provide more context, enabling the model to make more informed decisions about building changes.

**Figure 2:**
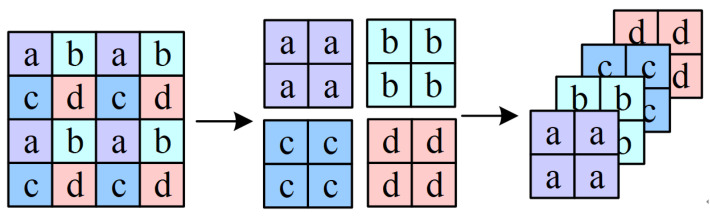
Patch merging process.

To generate multiscale features, as the network continues to deepen, the number of patches is reduced through the patch merging module. The given resolution is $\frac{H}{2^{i+1}}\times \frac{W}{2^{i+1}}\times C_{i}$ for i-th layer input. Through patch merging, the resolution becomes $\frac{H}{2^{i+2}}\times \frac{W}{2^{i+2}}\times C_{i+1}$ for the *i* + 1-th layer input. Four times the combination rate was used in the first stage, and two times the combination rate was used in the next three stages. The latter three stages are shown as an example in Figure 2.

In this stage, we want to conduct merging at a doubled rate, so we select points every other point, which means that for this tensor, we select points a, a, a, and a each time. The a, b, c, and d items in the figure are not values in the matrix but rather ordinal numbers that we use to make this process easier for the reader to understand. Patches with the same ordinal number are then merged together. After this spaced sampling procedure, the original tensor becomes four tensors. The size of each tensor is $\frac{H}{2}\times \frac{W}{2}$. The tensors are then spliced in the dimension of C, and the tensor size becomes $\frac{H}{2}\times \frac{W}{2}\times 4C$, exchanging spatial dimensionality for a greater number of channels. This process is very similar to the pooling operation in a convolutional neural network. The image changes from the initial 4 × 4 × C to 2 × 2 × 4C. As the network continues to deepen, features of more scales are formed, and the following resolutions can be obtained: $\frac{H}{4}\times \frac{W}{4}$, $\frac{H}{8}\times \frac{W}{8}$, $\frac{H}{16}\times \frac{W}{16}$, and $\frac{H}{32}\times \frac{W}{32}$.

#### 2.2.2. Siamese-Layered Transformer Block

**Figure 3:**
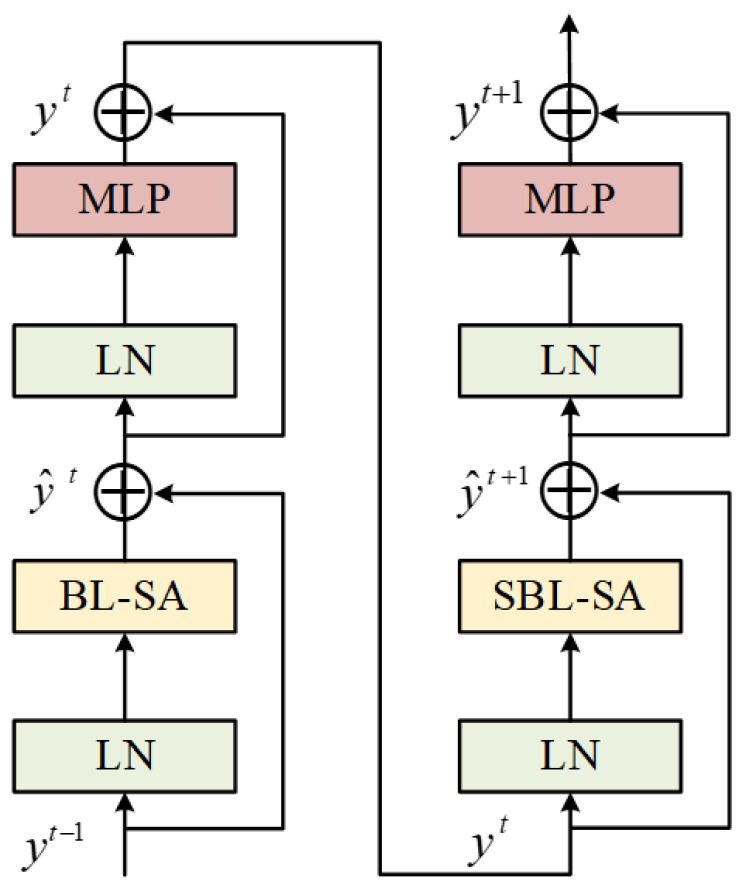
Transformer block structure.

In the Siamese transformer architecture, each part has four transformer blocks, and each layer of the transformer can generate building features at different scales. The input patches of each layer are processed through patch merging and transformer blocks to generate building features at different scales. These four components produce features with sizes of $\frac{H}{4}\times \frac{W}{4}$, $\frac{H}{8}\times \frac{W}{8}$, $\frac{H}{16}\times \frac{W}{16}$, and $\frac{H}{32}\times \frac{W}{32}$. The layered features consist of both high-resolution coarse-grained features and low-resolution fine-grained features, and these features are utilized in pairs. The features at different levels serve as input to the difference module for further learning of differential features. The standard transformer architecture and its processing on images both use global attention, and the relationship between one patch and all other patches is calculated. Globally, a complexity equivalent to the square of the number of patches is incurred. For better supporting the application device and for the sake of our training work, we crop the input image to 256×256 pixels as the initial input values for the model. The length of the patch input sequence in the first layer is $\frac{256}{4}\times \frac{256}{4}=4096$. The computational complexity of using such a sequence length as a global self-attention mechanism is usually very high. Therefore, the block-level scope-based method is used to calculate the self-attention. The initial stage is chosen as an example below. The dimension of the feature tensor is $\frac{H}{4}\times \frac{W}{4}\times C$, which is divided into a number of small scopes, each with N×N patches, and self-attention is performed for each scope, effectively reducing the computational complexity. Sequence length is effectively reduced. Although a lower computational cost is achieved through the block-level scope self-attention mechanism, the scopes are isolated, and the advantage of using the transformer’s global modeling ability is lost. Therefore, after using the block-level scope self-attention mechanism, a scope move operation is performed to enable information interaction between block-level scopes. As the network continues to deepen, this method can achieve approximate global modeling. Figure 3 shows the transformer block structure.

In Figure 3, BL-SA represents the self-attention module based on block-level scopes, SBL-SA represents the translation operation conducted on the block-level scopes, which are used to better achieve the effect of global modeling, and the MLP layer is used to perform nonlinear transformations and mappings on features.

The calculation process of the entire transformer block is as follows:

(1) $${\hat{y}}^{t}=BL-SA\left(LN\left(y^{t-1}\right)\right)+y^{t-1}$$

(2) $$y^{t}=MLP\left(LN\left({\hat{y}}^{t}\right)\right)+{\hat{y}}^{t}$$

(3) $${\hat{y}}^{t+1}=SBL-SA\left(LN\left(y^{t}\right)\right)+y^{t}$$

(4) $$y^{t+1}=MLP\left(LN\left({\hat{y}}^{t+1}\right)\right)+{\hat{y}}^{t+1}$$

Equation (1): This equation represents the self-attention (SA) module in a regular block-level scope configuration. It applies layer normalization (LN) to the input features $y^{t-1}$, performs self-attention computations using the BL-SA module, and then sums the residual connections $y^{t-1}$ derived from the previous layer.

Equation (2): This equation involves applying a multilayer perceptron (MLP) with GELU nonlinearity to the BL-SA module output. Layer normalization is applied again, and the result is summed with the output ${\hat{y}}^{t}$ of the BL-SA module via a residual connection.

Equation (3): This equation represents the self-attention (SBL-SA) module with a sliding block-level scope. It applies layer normalization to the output $y^{t}$ of the previous MLP layer, performs self-attention computations using the SBL-SA module with a sliding configuration, and sums the residual connections $y^{t}$ derived from the previous layer.

Equation (4): This equation involves applying a multilayer perceptron (MLP) with GELU nonlinearity to the SBL-SA module output. Layer normalization is applied, and the result is summed with the output of the SBL-SA module possessing ${\hat{y}}^{t+1}$ through a residual connection.

${\hat{y}}^{t}$ and $\;y^{t}$ represent the outputs of the block-level self-attention (BL-SA) and multilayer perceptron (MLP) modules and the characteristic outputs of the SBL-SA and MLP modules, respectively.

#### 2.2.3. Difference Module

Metric-based deep learning has been applied in a wide range of remote sensing applications, demonstrating improved performance in change detection methodologies rooted in metric learning. In the context of metric-based deep learning, a network is guided to acquire the nonlinear mapping from the input to the embedding space. In this embedded vector space, akin or unaltered samples are close to one another, whereas disparate or evolving samples are distributed at a greater distance from each other.

Four difference modules are used to calculate the multilevel difference of the changed images before and after the layered transformer encoder. In this paper, the role of the difference module is to learn the optimal metric of the difference characteristics of buildings at each scale during the training process.

In remote sensing-based image building change detection tasks, the feature maps $F_{before}^{i}$ and $F_{after}^{i}$ give the multiscale features of a building at a certain level. Each pixel in these feature maps contains information about the corresponding position in the image. When calculating the differences between feature maps, usually, the pixels at the corresponding locations are subject to difference calculations. The Euclidean distance is a method that is used to measure the straight-line distance between two points. For each corresponding pixel value at each position (pixel), the Euclidean distance is calculated as follows. For two feature maps $F_{before}^{i}$ and $F_{after}^{i}$, at the same position (the corresponding pixel position), their pixel values are taken as $P_{before}$ and $P_{after}$. The Euclidean distance at the corresponding position is calculated with the following formula: $Euclidean\;Distance=\sqrt{{\left(P_{before}-P_{after}\right)}^{2}}$. This calculated Euclidean distance constitutes a single pixel value of the difference feature map *D*. The above steps are repeated to iterate through all the pixels of the feature map for obtaining the complete difference feature map *D*.

The difference feature map *D* reflects the feature differences exhibited by the building at different points in time, and the computation of the Euclidean distance helps the model learn to differentiate between neighboring and nonneighboring pixels via the contrast loss during the training phase, thus improving the accuracy of the change detection process.

Given the multiscale feature map of the *i*-th layer $F_{before}^{i}$, $F_{after}^{i}$, the Euclidean distance between the feature map pixels is calculated to generate the difference distance map $D\in R^{H_{i}\times W_{i}}$, where $W_{i}$ and $H_{i}$ represent the width and height of the input image at the *i*-th layer, respectively. In the training stage, the contrastive loss is used to learn the network parameters so that the adjacent pixels are clustered, while the nonadjacent pixels are scattered. Then, the change map is derived by applying a consistent threshold value $F_{change}^{i}$.

(5) $$F_{change}^{i}\left(n,m\right)=\left\{\begin{array}{cc} 1 & D_{\left(n,m\right)}>\theta \\ 0 & else \end{array}\right.$$

where *n* and *m* represent the width and height, respectively. θ is a fixed threshold to distinguish change regions.

In the domain of change detection through remote sensing images, there is a significant disproportion in the quantities of altered and unaltered samples. Often, the altered pixels constitute only a small fraction of the total, and during training, there is a potential bias introduced into the network. To address this class imbalance issue, we employ a batch-balanced comparative loss. This method leverages batch weight priors to adjust the category weights within the original contrastive loss. Given a set of bitemporal samples in a batch $\left(X^{*(1)},X^{*(2)},M^{*}\in R^{B\times H_{i}\times W_{i}}\right)$, a set of distance maps is derived from the difference module $D^{*}$. Here, *B* denotes the batch size, and $M^{*}$ represents a collection of binary label maps within a batch. The batch balanced comparative loss function can be mathematically expressed as follows:

(6) $$\begin{array}{c} L\left(D^{*},M^{*}\right)=\frac{1}{2}\frac{1}{n_{u}}\sum_{b,n,m}\left(1-M_{b,n,m}^{*}\right)D_{b,n,m}^{*}+\frac{1}{2}\frac{1}{n_{c}}M_{b,n,m}^{*}Max\left(0,k-D_{b,n,m}^{*}\right) \end{array}$$

where the subscripts *b*, *n*, and *m* represent the batch size, width, and height, respectively. If the parametric distance between changed pixel pairs exceeds the threshold value *k*, it will not impact the loss function. For this particular task, the threshold *k* is set to 2. The threshold “*k*” acts here as a sensitivity parameter for determining the magnitude required for a change to be considered significant. The reason for setting the threshold to 2 is to ensure that the model is able to focus on capturing relatively large changes in the feature difference maps, which are more likely to correspond to substantial changes in the corresponding remotely sensed images. This setting helps the model distinguish between significant changes and noise in the feature difference maps, thus improving the performance of the model. Adjusting the threshold value “*k*” can significantly affect the ability of the model to detect changes in remotely sensed images. Higher thresholds may make the model more conservative, as they will focus on substantial changes and reduce the likelihood of false alarms; however, they may also miss subtle changes. Conversely, lower thresholds may increase the sensitivity of the model, allowing it to capture smaller changes; however, the model may also introduce additional noise and false alarms. $n_{u}$ and $n_{c}$ represent the counts of nonchanged and changed pixel pairs, respectively. These counts can be computed by summing the corresponding class labels.

(7) $$n_{u}=\sum_{b,n,m}1-M_{b,n,m}^{*}$$

(8) $$n_{c}=\sum_{b,n,m}M_{b,n,m}^{*}$$

### 2.3. Decoder

In this paper, a decoder with a streamlined design is employed to combine difference maps of multiscale building features for change prediction. The proposed decoder obtains the fused difference feature maps by up-sampling and feature fusion and again undergoes the inverse convolution to obtain the difference feature maps of the original image size, and finally, it obtains the segmentation results. This simplified decoder, consisting of only MLP layers, skillfully avoids the handcrafted and highly computationally demanding components that are common in other approaches. The successful realization of this simple decoder is due to three key factors. First, our hierarchical transformer encoder has a larger effective sensing field than those of other methods and includes more than traditional CNN encoders. Second, the consistent designs of the decoder and encoder, using the same MLP and SBL-SA modules, drive the model to be more uniform and efficient during the training and inference processes. Finally, by simplifying the model structure, the complexity of the overall architecture is reduced, making the model easier to understand and debug while improving its scalability for various tasks and application scenarios.

#### 2.3.1. Feature Fusion

**Figure 4:**
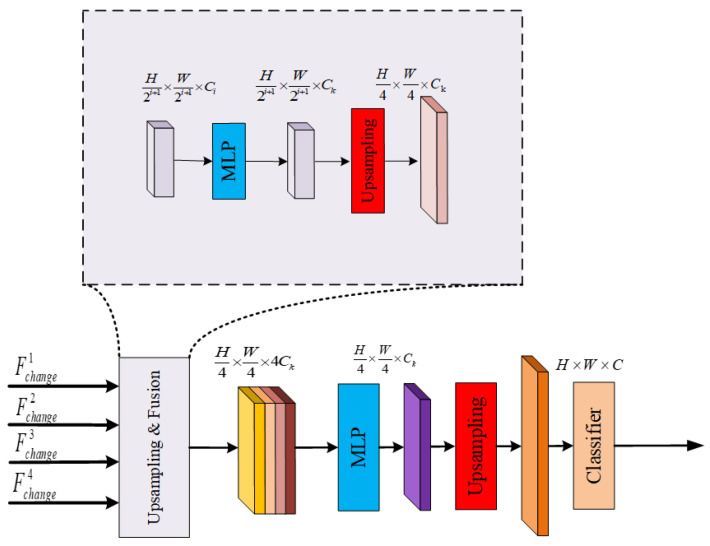
Feature fusion process.

Building change detection in remote sensing images is an intensive detection task in which multiscale feature fusion has a clear advantage. The target objects may have different sizes and scales. However, multiscale feature fusion enables the model to process the features of multiple scales simultaneously and thus better detect objects with different sizes. In addition, multiscale feature fusion helps capture richer contextual information at different feature levels, enabling the model to better differentiate target objects from the background in the dense detection task. Finally, multiscale feature fusion helps to improve the robustness of the model to transformations such as scale change, rotation, and occlusion, making multiscale feature fusion an important technique in dense detection tasks. The entire feature fusion process is shown in Figure 4.

In Figure 4, a feature fusion process is presented for predicting changes in multiscale building features. First, a multiscale feature difference map is obtained from the encoder, marking the beginning of the fusion process. Next, each multiscale feature disparity map is processed through the MLP layer to unify the channel dimensions. Subsequently, each feature disparity map is upsampled and resized to H/4 ×W/4. Finally, the upsampled feature disparity maps are spliced and fused in the MLP layer to obtain the final fusion result for the feature disparity maps. This process is expressed as follows:

(9) $${\tilde{F}}_{change}^{i}=Linear\left(C_{i},C_{k}\right)\left(F_{change}^{i}\right)\forall i$$

(10) $$\begin{array}{c} {\hat{F}}_{change}^{i}=Upsample\left(\left(H/4,W/4\right),bilinear\right)\left({\tilde{F}}_{change}^{i}\right) \end{array}$$

in which $C_{k}$ represents the embedding dimension. In Equation (9), the linear operation denotes a linear transformation of the feature map, which is usually understood as a fully connected layer or linear projection layer, transforming the input feature map to a new feature space. This transformation is crucial for unifying the channel dimensions of the feature maps, ensuring the compatibility of the subsequent processing and fusion steps. The upsampling process in Equation (10) involves the use of bilinear interpolation to increase the spatial resolution of the feature map. Bilinear interpolation is chosen for upsampling because it effectively preserves the overall structure and details of the feature map while increasing its dimensions. This interpolation method calculates new pixel values by computing the weighted average of the four nearest pixels in the original feature map, resulting in a smooth and visually coherent upsampled image. Alternatives to bilinear interpolation include nearest-neighbor interpolation, bicubic interpolation, and transposed convolution (also known as inverse convolution). Nearest-neighbor interpolation simply copies the nearest pixel values to fill in new pixels, which can lead to blocky artifacts and detail losses. Bicubic interpolation provides higher-quality information than does bilinear interpolation but requires additional computational resources.

Finally, the upsampled feature difference maps are spliced and fused in the MLP layer, thus obtaining the fusion results of the four feature difference maps. Then, S = 4 and K = 3. The deconvolutional layer operation, which maps the fused features to F upsampling to the same size as the original image H×W. This process is expressed as follows:

(11) $$\begin{array}{c} F=Linear\left(4C_{k},C_{k}\right)\left(Cat\left({\hat{F}}_{change}^{1},{\hat{F}}_{change}^{2},{\hat{F}}_{change}^{3},{\hat{F}}_{change}^{4}\right)\right) \end{array}$$

(12) $$\hat{F}=\mathrm{Conv}Transpose2D\left(S=4,K=3\right)\left(F\right)$$

#### 2.3.2. Segmentation Results

The differentially fused feature map is obtained through feature fusion. Subsequently, a deconvolution operation is employed to align the size of the feature map with that of the original input image. The fused features are finally processed through another MLP layer, and the prediction resolution is $H\times W\times N_{cls}$, where $N_{cls}\left(=2\right)$ is the number of classes, that is, changing and not changing. This process can be expressed as:

(13) $$Mask=Linear\left(C_{k},N_{cls}\right)\left(\hat{F}\right)$$

## 3. Experimental Part

### 3.1. Dataset Introduction

Two public datasets are used in the experiments, namely, LEVIR-CD [24] and WHU-CD [25].

LEVIR-CD is an extensive dataset of remote sensing images tailored for the purpose of change detection. This dataset is usually used to analyze the changes in surface characteristics between different time points, such as building changes, urban sprawl, and river changes. The LEVIR-CD dataset contains 637 annotated image pairs, and each image pair contains two high-resolution remote sensing images of Google Earth at times t1 and t2. These images have a resolution of 1024×1024 pixels, full-color (RGB), covering different regions and seasons to show different types of surface changes, such as urban expansion and building demolition. In this paper, these images are cropped to 256×256 nonoverlapping small blocks and randomly divided into three parts to obtain 7120/1024/2048 samples in the training, validation, and test sets.

The Wuhan University Change Detection (WHU-CD) dataset is a general remote sensing image change detection dataset. The WHU-CD dataset is provided by the China Geographic Information Science Research Center of Wuhan University. This dataset comprises a pair of high-resolution remote sensing images, featuring a spatial resolution of 0.075 m and a size of 32,507 × 15,354. This image pair represents two remote sensing images at different time points. These images cover a variety of terrains, such as urban buildings, farmlands, mountains, and rivers, and demonstrated various surface change phenomena, such as land use change and urban sprawl. These images are cropped to 256×256. For the nonoverlapping small blocks, 6096/762/762 samples were obtained for the training, validation, and test sets.

### 3.2. Experimental Settings

The experiments in this paper were performed on a Windows 10 (64-bit) operating system with an 11th generation Intel(R) Core(TM) i9-9900K @ 3.60 GHz processor, an NVIDIA GeForce RTX 2060Ti (16 GB) graphics processing unit (GPU), and the Python 3.8 environment. Conventional data augmentation, including random rescaling (0.8–1.2) and Gaussian blurring, are applied to the data (image patch). A 5 × 5 Gaussian kernel is used for Gaussian blurring, which is set to a standard deviation of 1.5 to balance image blurring and detail preservation. The optimization of the model employed stochastic gradient descent with momentum. A momentum value of 0.99 and weight decay of 0.0005 were set. The initial learning rate was established at 0.01 and progressively decreased in a linear fashion until reaching 0. The total number of iterations was 200, and a batch size of 16 was used to train the model.

### 3.3. Evaluation Indicators

The choice of performance evaluation metrics in this study is pivotal for gauging the proficiency of the proposed model in terms of detecting building changes in remote sensing images. These metrics offer numerical assessments of the model’s capacity to precisely recognize and categorize changes in an urban environment. The significance of these performance evaluation metrics is elucidated as follows:

(1) Precision: Precision assesses the accuracy of the model in identifying changed areas in the context of detecting building changes. It measures the proportion of true-positive predictions among all positive predictions made by the model. A high precision value indicates that the model has a low false-alarm rate, which is crucial for applications where the precise identification of building changes is essential.

(2) Recall: Recall measures the proportion of true-positive predictions relative to all actual positive instances in the input dataset. In the context of building change detection, recall signifies the ability of the model to identify all changed areas while minimizing false-negative instances. A higher recall value suggests that the model effectively captures the majority of the actual changes in the urban environment.

(3) F1 score: The F1 score represents the harmonic mean of precision and recall, offering a balanced assessment of the overall performance achieved by the model. It considers both false positives and false negatives, making it a valuable metric for comprehensively gauging performance. A high F1 score implies that the model achieves both precise identification and comprehensive coverage of changed areas, striking a balance between change detection accuracy and inclusiveness in terms of recognizing changes.

(4) Intersection over union (IoU): The IoU assesses the overlap between the predicted change areas and the actual ground-truth change areas. The calculation process involves dividing the intersection of the predicted and ground-truth areas by their union. The IoU offers insights into the spatial alignment and accuracy of the predictions produced by a model concerning the real changes occurring in an urban environment.

(5) Overall accuracy (OA): The OA evaluates the overall correctness of the predictions output by the model across all categories, offering a comprehensive assessment of the model performance. It serves as a fundamental metric for assessing the ability of the model to accurately classify areas both with changes and without changes. In this paper, the variation category F1 was used as the main evaluation indicator. The F1 is calculated based on the test accuracy precision and recall, and the calculation formula is as follows:

(14) $$F1=\frac{2}{reacll^{-1}+precision^{-1}}$$

The definitions of the precision, recall rate, intersection over union (IoU), and overall accuracy (OA) are given as follows:

(15) precision=TP/(TP+FP)

(16) recall=TP/(TP+FN)

(17) IoU=TP/(TP+FN+FP)

(18) OA=(TP+TN)/(TP+TN+FN+FP)

In this scenario, TP signifies the count of correctly identified positive samples, FP denotes the tally of erroneously reported negative samples, TN represents the number of accurately identified negative samples, and FN indicates the quantity of undetected positive samples.

### 3.4. Model Comparison Experiment

In this section, the performance of the proposed model in detecting building changes in remote sensing images is assessed through a comparison with several state-of-the-art methods.

FC-Siam-Di [26] (2018): A feature-level fusion method that employs a Siamese FCN to extract multilevel features and integrates dual-temporal information through the use of feature differences.

FC-Siam-Conc [26] (2018): A feature-level fusion method that utilizes Siam-FCN to extract multilevel features and employs feature stitching for integrating dual-temporal information.

FC-EF [26] (2018): An image-level fusion method that involves feeding a dual-temporal image as a single input into a fully convolutional neural network.

DTCDSCN [7] (2020): A multiscale feature stitching method that enhances the deep Siamese FCN by incorporating channel attention and spatial attention, aiming to obtain more discriminative features.

BIT [27] (2021): A transformer-based method that uses the transformer’s global modeling capability to perform feature differentiation through semantic tokens. Enhancing the contextual information in a convolutional neural network leads to a better change map.

ISNet [28] (2022): By combining margin maximization and targeted attention mechanisms, ISNet successfully enhanced separability in remote sensing image change detection, leading to superior performance.

Fusion-Former [29] (2023): A network that combines Transformer and CNN, Fusion-Former can fully leverage their respective advantages to achieve multi-scale information integration and feature extraction, thereby enhancing the accuracy and efficiency of change detection.

**Figure 5:**
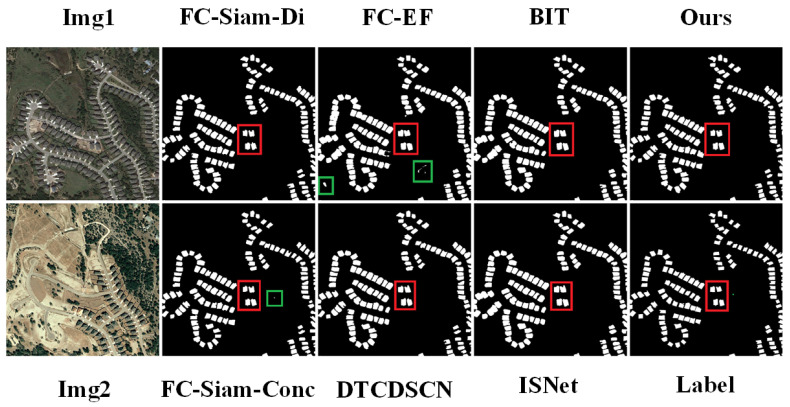
Comparison and visualization results of LEVIR-CD using different change detection methods.

**Figure 6:**
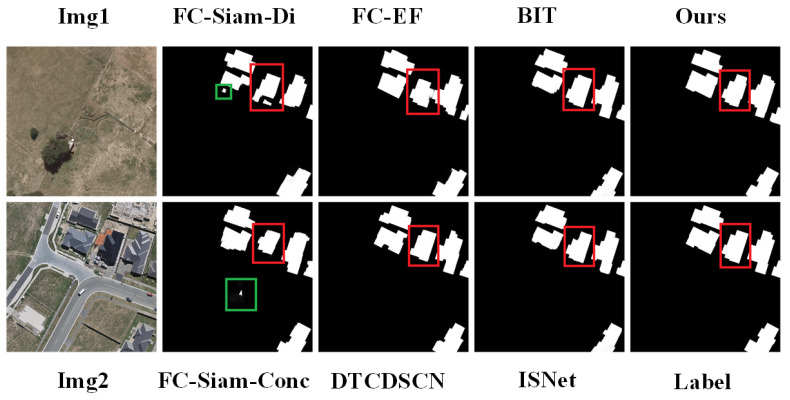
Comparison and visualization results of WHU-CD using different change detection methods.

Visual comparisons of these methods on the two datasets are depicted in Figure 5 and Figure 6. The model proposed in this paper outperforms the other models in terms of the visualization results. The proposed model can better avoid false-positives (shown by the green box in the figure), and this type of erroneous detection can be reduced by enhanced feature discrimination based on global context modeling. The building boundary information is critical for building change detection. Better multiscale difference features lead to better performance in processing some boundary information of buildings (indicated by the red box in the figure).

The overall comparison results of the LEVIR-CD and WHU-CD test sets are shown in Table 1. The quantitative results show that the model proposed in this paper outperforms other methods on these two datasets. For example, on the LEVIR-CD dataset, the proposed model achieves F1 scores higher than those of other comparative models except for being on par with ISNet. On the WHU-CD dataset, the overall performance of the proposed model is also superior to that of other comparative models. By measuring the multiscale difference feature information and fusing low-level features with high spatial accuracy and high-level semantic features to complete the pixel-level prediction task, the proposed model achieves superior performance. The context is modeled in the highly abstract global space–time scope, and the context is used to enhance the feature representation in the pixel space.

In comparison with state-of-the-art models, the proposed model demonstrates significant advantages in overall performance. Here is a comparative analysis of them.

For BIT using a CNN backbone network (ResNet), high-level semantic features are extracted from pairs of input images. Each temporal feature map is then transformed into a compact set of semantic tokens using spatial attention. Subsequently, a transformer encoder is employed to model the context within the two token sets. The context-rich tokens generated are then reprojected into pixel space by a Siamese-transformer decoder to enhance the original pixel-level features. Finally, feature difference images are computed from the two refined feature maps and input into a shallow CNN to produce pixel-level change predictions. Although it does not incorporate specific modules designed for change detection, its strong performance is attributed to its superior global modeling capabilities and effective enhancement of original features. However, BIT does not acquire multi-scale features like the proposed model, which may have some impact on handling boundary information, potentially leading to slight blurring at boundaries and a small number of false positives. Therefore, its performance is slightly inferior to the proposed model.

ISNet introduces a margin maximization module to learn and clarify the difference between changing and invariant semantics, alleviating the issue of blurred boundaries between hierarchical features during feature extraction. The Targeted Arrangement of Attention Mechanisms is a plug-and-play attention module. In the feature extraction stage, the Channel Attention module is inserted into each stage to emphasize specific channels in the feature maps by learning different weights for each channel, promoting semantic-specific feature extraction. In the multi-scale feature-fusion stage, the Spatial Attention highlights the positional change response in the fused bi-temporal features. ISNet adopts a top-down fusion path to merge multi-scale features, similar to the hierarchical transformer processing concept proposed in this model. By obtaining features at different scales through multiple backbone blocks and then fusing these features, it can better handle building boundary information. ISNet slightly lags behind the proposed model in global modeling capability. Models that capture long-distance dependencies and global features can better capture subtle changes and overall characteristics in images, thereby improving the F1 score. Therefore, the F1 score of the proposed model is better than ISNet.

Fusion-Former combines the characteristics of Transformer and Convolutional Neural Networks (CNN) for building change detection. By introducing the Fusion-Block, it implements the self-attention mechanism of the transformer and the bidirectional interaction of CNN depth convolution, enhancing the modeling capability in channel and spatial dimensions. This enables the integration of multi-scale information and feature extraction. On the WHU-CD dataset, the recall and F1 scores of Fusion-Former are slightly higher than those of the proposed model, which may be attributed to the Vision-Module. This module can more effectively encode fine-grained information, enhancing the recognition ability of change regions and thus improving the model’s detection capability for change regions. However, Fusion-Former has some shortcomings [29] in edge feature extraction, and the fusion of Transformer and CNN also makes the model structure more complex. In contrast, the proposed model combines the difference module that captures the difference features before and after building changes with the Siamese transformer-based structure, which can better extract multi-scale features of buildings in bitemporal remote sensing images, facilitating the handling of building boundary information. This paper only utilizes the transformer structure, making the overall model structure more concise and unified.

In summary, compared to other state-of-the-art models, the proposed model demonstrates significant advantages in overall performance. In the comparative analysis, Fusion-Former achieves slightly higher recall and F1 scores in some aspects but suffers from shortcomings in edge feature extraction and the complexity of the model structure. In contrast, ISNet and BIT lag behind in global modeling capability and multi-scale feature extraction compared to the proposed model. Overall, the proposed model exhibits superior performance in handling building change detection tasks, attributed to its concise and unified model structure, as well as its effective handling of boundary information, making it more reliable.

These qualitative and quantitative comparisons demonstrate that the proposed change detection method outperforms the state-of-the-art detection methods in the following Table 1. These better results are mainly obtained because by utilizing the Siamese architecture, transformer encoder, difference module, and decoder together, the model proposed in this paper is able to better capture multiscale disparity features, merge low-level features with high-level semantic features, and complete the task of change detection at the pixel level, thus achieving superior performance.

### 3.5. Ablation Experiment

To further elucidate the effectiveness of the model proposed in this paper, ablation experiments were performed on the LEVIR-CD and WHU-CD datasets. Three ablation experiments were designed:

(1) The Siamese architecture is used to layer the transformer encoder. This model is designed to investigate the contribution of the Siamese architecture to the performance of the proposed model. The Siamese architecture is used to extract features reflecting differences at multiple levels in dual-temporal remote sensing images. By using the Siamese architecture to layer the transformer encoder, the model can capture long-distance spatial dependencies and better process building boundary information.

(2) The Siamese architecture is used to layer the transformer encoder and the difference module. This model is designed to investigate the contribution of the difference module to the performance of the proposed model. The difference module is used to obtain multiscale difference feature representations, and richer context information is captured at different feature levels through feature fusion. By using the Siamese architecture to layer the transformer encoder and the difference module, the model can better characterize the change characteristics and reduce the false-positive rate.

(3) The Siamese architecture, transformer encoder, and difference module are used in cooperation with the decoder: This model is designed to investigate the contribution of the decoder to the performance of the proposed model. The decoder is used to upsample the feature maps and generate the final change detection map. By using the Siamese architecture, transformer encoder, and difference module in cooperation with the decoder, the model can better process building boundary information and improve its change detection performance.

**Figure 7:**
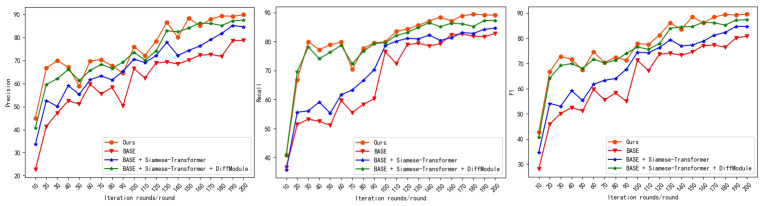
Ablation experiments on the LEVIR-CD dataset.

**Figure 8:**
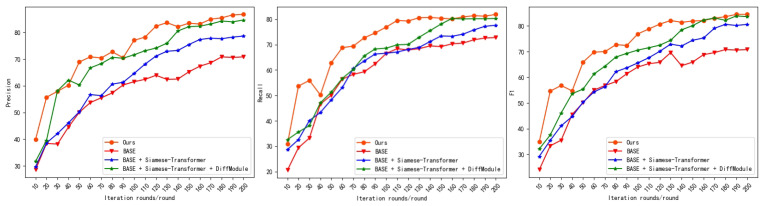
Ablation experiments on the WHU-CD dataset.

In these figures, the model iterations are depicted on the horizontal axis, while the precision, recall, and F1 values of the model are represented on the vertical axis for the three ablation experimental models and the proposed model. Figure 7 and Figure 8 show that in the ablation experiments, the approach introduced in this paper exhibits superior accuracy during training and achieves a more effective final convergence based on the Siamese architecture and the differential module model.

In the ablation experiments, three different experimental models are designed to investigate the contributions of specific modules to the overall performance of the proposed model. The quantitative metrics used to evaluate the performance of each model include precision, recall, and the F1 score. The results of the ablation experiments are presented in Table 2 and Table 3, which show that the proposed model outperforms the other models on both the LEVIR-CD and WHU-CD datasets in terms of precision, recall, and the F1 score. Specifically, the F1 scores of the proposed model are 8.7%, 4.9%, and 2.1% greater than those of the other models on the LEVIR-CD dataset and 12.1%, 2.9%, and 1.2% greater on the WHU-CD dataset. The main reason for this finding is that the hierarchical feature maps generated by the Siamese architecture together with the difference module can better characterize the observed change characteristics, thus reducing false positives. Moreover, multilevel and multiscale difference features can better process building boundary information, thus enhancing the model performance.

**Figure 9:**
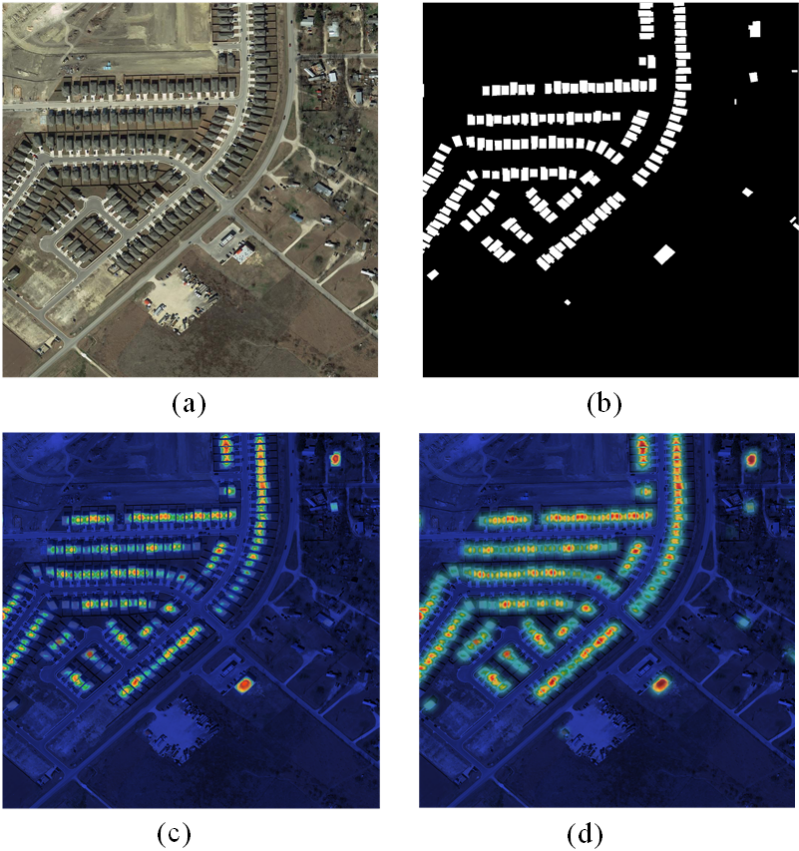
LEVIR-CD attention maps: (**a**) original image; (**b**) label; (**c**) baseline model; and (**d**) proposed model.

**Figure 10:**
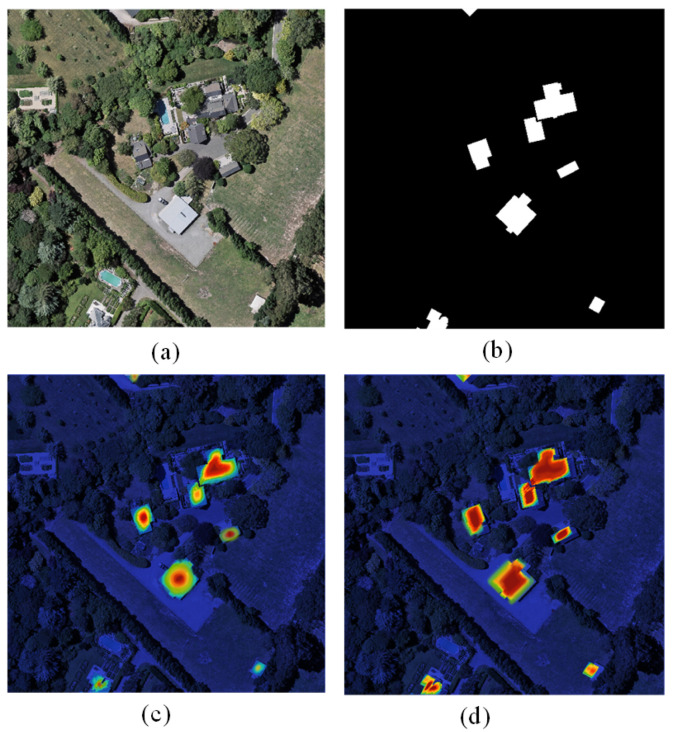
WHU-CD attention maps: (**a**) original image; (**b**) label; (**c**) baseline model; and (**d**) proposed model.

To enhance the visualization and analysis of the experimental outcomes, feature visualizations using the proposed method and the baseline are presented in Figure 9 and Figure 10. The visualizations in these figures were assessed using the same model as those used in the corresponding rows in Figure 5 and Figure 6. In these visual representations, the highlighted regions signify the pivotal regions of feature emphasis. The model places increased emphasis on the identified change region and demonstrates superior handling of boundary information details. As shown in Figure 9c,d in Figure 10 and Figure 10c,d compared with the feature focus of the baseline, the present network model pays more attention to the foreground and global information, thus obtaining finer content details and building boundary information. In addition, these features’ attention highlights the locations of foreground change regions in complex scenes, thus improving the change detection performance.

The attention maps shown in Figure 9 and Figure 10 are computed using the proposed model. These attention maps highlight the regions of the input image that the model considers most important for making its decision. Specifically, the attention maps are generated by computing the attention weights of each feature map in the encoder of the model. The attention weights are subsequently used to weight the feature maps, resulting in a weighted sum that represents the most important regions of the input image. The significance of these attention maps lies in their ability to provide insights into the decision-making process of the model. By visualizing the regions of the input image that the model considers most important, we can gain a better understanding of the features and patterns that the model is using to make its decision. This approach can be particularly useful for identifying areas where the model may make errors or for identifying areas where the model is particularly effective.

## 4. Conclusions

This paper puts forward a Siamese transformer network that incorporates a difference module, aiming to detect changes in buildings within remote sensing images. The proposed Siamese layered transformer architecture is employed to extract features reflecting differences at multiple levels in dual-temporal remote sensing images. The difference module was used to obtain multiscale difference feature representations, and richer context information was captured at different feature levels through feature fusion. Finally, the change detection result was obtained. In this mechanism, the feature representations at different scale levels were obtained through Siamese multilayer transformers, which capture the long-distance spatial dependence and better process the building boundary information. In addition, the difference module compares the difference maps of the dual-temporal images to learn a better representation, effectively reducing the false-positive information in the dual-temporal remote sensing images.

On the LEVIR-CD dataset, the proposed method achieves an F1 score of 89.58%, surpassing the F1 scores of other state-of-the-art methods, including FC-EF (83.38%), FC-Siam-DI (86.18%), FC-SiamConc (83.66%), DTCDSCN (87.65%), BIT (89.29%), ISNet (89.58%), and Fusion-Former (89.53%). Similarly, on the WHU-CD dataset, the proposed method attains an F1 score of 84.51%, surpassing the vast majority of comparative methods, such as FC-EF (69.32%), FC-Siam-DI (58.99%), FC-SiamConc (66.64%), DTCDSCN (71.96%), BIT (83.98%), and ISNet (83.24%), but is slightly inferior to Fusion-Former (86.00%). Additionally, the proposed method demonstrates superior performance in terms of the precision, recall, IoU, and overall accuracy metrics on both utilized datasets when compared to other methods. The comparative analysis highlights the effectiveness of the proposed method, particularly in terms of handling complex urban environments and subtle changes in remote sensing imagery. In these scenarios, the proposed method exhibits outstanding performance compared to that of the existing methods.

While the proposed model demonstrates excellent performance, a potential limitation for future research to address lies in its ability to handle complex or dynamic environmental conditions, such as variations in lighting, weather, or seasons. Subsequent studies can focus on enhancing the robustness of the model to these environmental factors. We plan to collect and curate a dataset for detecting building changes from unmanned aerial vehicle (UAV) remote sensing images. This dataset will be used to adjust the model training process, and its applicability will be extended to scenarios such as environmental monitoring and urban planning. This extension will aim to further assess the generality and adaptability of the developed network.

## Figures and Tables

**Table 1:** Quantitative results of LEVIR-CD and WHU-CD by different change detection methods. * Presents the results reported in the original article.

Method	LEVIR-CD					WHU-CD				
	Pre.	Rec.	F1	IoU	OA	Pre.	Rec.	F1	IoU	OA
FC-Siam-Di	89.42	83.16	86.18	75.51	98.65	47.56	77.61	58.99	41.70	95.65
FC-Siam-Conc	91.89	76.78	83.66	71.93	98.41	60.91	73.57	66.64	49.93	97.03
FC-EF	86.88	80.16	83.38	71.51	98.36	71.60	67.19	69.32	53.06	97.56
DTCDSCN	88.52	86.80	87.65	78.04	98.75	63.93	82.30	71.96	56.18	97.43
BIT	89.23	89.35	89.29	80.65	98.90	86.62	81.47	83.98	72.38	98.75
ISNet	91.37	87.86	89.58	80.68	98.89	84.83	81.71	83.24	72.47	98.38
Fusion-Former *	90.30	88.78	89.53	81.05	-	86.40	86.72	86.00	72.86	-
Ours	89.99	89.18	89.58	80.69	98.98	86.71	81.92	84.51	72.87	98.86

**Table 2:** Quantitative results of the effect of different modules on the LEVIR-CD dataset.

Method	Pre.	Rec.	F1	IoU	OA
Our	89.9	89.1	89.5	80.6	98.9
Base	78.8	82.8	80.8	71.6	96.4
Base+Siamese-Transformer	84.6	84.6	84.6	75.2	98.1
Base+Siamese-Transformer+ DiffModule	87.6	87.3	87.4	79.3	98.6

**Table 3:** Quantitative results of the influence of different modules on the WHU-CD dataset.

Method	Pre.	Rec.	F1	IoU	OA
Our	86.7	81.9	84.5	72.8	98.8
Base	73.1	71.8	72.4	60.3	94.5
Base+Siamese-Transformer	83.3	79.9	81.6	68.6	97.5
Base+Siamese-Transformer+ DiffModule	85.6	81.3	83.3	71.5	97.9

## Data Availability

The data is available at http://gpcv.whu.edu.cn/data/building_dataset.html, https://chenhao.in/LEVIR/ (accessed on 10 January 2024) and the model code can be provided upon request from the corresponding author, Feng Liu.
